# Growth trends analysis of unilateral condylar hyperplasia followed up with planar scintigraphy: Retrospective overview of 249 cases

**DOI:** 10.1097/MD.0000000000028226

**Published:** 2021-12-23

**Authors:** Pingan Liu, Jun Shi

**Affiliations:** a Department of Nuclear Medicine, Ninth People's Hospital, Shanghai Jiao Tong University School of Medicine, Shanghai, China; b Department of Oral and Craniomaxillofacial Science, Ninth People's Hospital, Shanghai Jiao Tong University School of Medicine, Shanghai, China.

**Keywords:** bone scintigraphy, condylar hyperplasia, MDP ;

## Abstract

The current research aimed to retrospectively investigate the trends of the growth of condylar hyperplasia with serial planar scintigraphs.

Patients of unilateral condylar hyperplasia with at least one follow-up planar scintigraph were retrospectively included in the study. Patients’ age, gender at the initial scan, durations of following scans, and ratios between condylar activities were recorded.

The study retrospectively included 111 patients of unilateral condylar hyperplasia. Patients were divided into 3 groups (progressive, relatively stable, regressive) according to ratio variation between initial and last scans. There were 23 (21%) patients fell into the progressive group, 40 (36%) patients into the relatively stable group, and 48 (43%) patients into the regressive group. More female patients were in the progressive group than those in the other groups (*P* < .01). There were no significant differences among the 3 groups in terms of age or durations of follow-up (*P* > .05). There were no strong relations between ratio differences and ages. However, a weak relation seems to exist in the regressive group with *r* = −0.240, (*P* = .10).

Our investigation showed that more than a half of patients with condylar hyperplasia remain constantly or progressively active growth in patients in the follow-up scans. Roughly less than a half of patients showed regressive trends toward normal growth. Patients’ age seemly does not play a role in the growth trend pattern, although there are no optimum follow-up periods, regularly follow-up scans are needed to determine the growth status of condylar hyperplasia.

## 1 Introduction

Unilateral condylar hyperplasia (UCH) is characterized by excessive pathologic growth in one of the mandibular condyles, which leads to facial asymmetry. The precise etiology of this condition is not clear.^[1]^ The treatment option for patients with clinically progressive mandibular asymmetry and hyperactive growth in the mandibular condyle is high condylectomy, which could arrest the progression of the condition.^[2]^ Conventional orthognathic procedures can be used to correct residual asymmetry once the hyperplasia is inactive. The assessment of UCH is critical to the clinical treatment decision. Planar bone scintigraphy with 99mTc-methylene diphosphonate (99mTc-MDP) is demonstrated to be effective to predict the presence of an ongoing condylar growth.^[3,4]^ The condyle is considered hyperactive if the difference in the regional activity between the right and the left condyle is greater than 10%. Recently, bone single-photon emission computed tomography (SPECT) or combined CT (SPECT/CT) is used to assess the UCH for better isolation of the activity of the mandibular condyle from that of its adjacent bone.^[5–8]^ Higher sensitivity and specificity have been documented with SPECT or SPECT/CT than that of planar scintigraphy in assessing active condylar hyperplasia. However, there is a strong positive correlation between uptake values on the SPECT and planar images for condyles.^[9]^ Bone scan with planar or SPECT image has incorporated into the clinical treatment decision-making process with UCH.^[6,9,10]^

However, patients with active UCH need to be followed up if a deferred surgery strategy is adopted. When will condyles cease growing is not known, and the optimal follow-up frequency is not clear, and these questions should be addressed. For planar bone scintigraphy used in clinical practice for many years since its inception and has accumulated huge valuable data which provide a rare opportunity to shed light on these aspects of condylar hyperplasia development. Therefore, we aimed to evaluate the changes in tracer uptake trend with the quantifications of bone scintigraphs in follow-up scans with planar bone scintigraphy.

## 2 Methods

### 2.1 Research design

This was a retrospective study approved by the institutional review board, and the informed consent was waivered.

### 2.2 Case selection

From our clinical database, image datasets were retrieved from patients of suspected active UCH who had undergone planar bone scintigraph between January 2009 and March 2017.

Inclusion criteria were that patients with planar bone scintigraph revealed active UCH with at least 1 follow-up planar bone scintigraph during the period were included in the analysis.

Exclusion criteria were: history of temporomandibular joint surgery; neoplastic pathology of the temporomandibular joint; systemic diseases that could potentially affect the temporomandibular joint; congenital conditions associated with facial asymmetry.

Patient's age, sex, initial scan data, and follow-up scan data were recorded.

### 2.3 Data acquisition and analysis

The patients were intravenously injected 555 to 851 MBq (15–23 mCi) 99mTc-MDP for imaging depending on the body weight (14.8 MBq/kg). Four hours later, static Images were acquired on a hybrid SPECT/CT dual-head gamma camera Inifinia Hawkeye 3 (GE Healthcare: Chicago, IL, USA), equipped with parallel-hole, low-energy, high-resolution collimators. Photo peak was set with 140 keV and a 20% symmetrical window, lateral planar images of condyles were acquired (256 × 256 matrix, 5 minutes per image).

### 2.4 Interpretation of planar images

Planar images were assessed by experienced nuclear medicine physicians. For planar images, precise regions of interest (ROIs) were drawn over the condyles. For planar image, ROI was drawn over one condyle in one lateral planar image and was copied and placed to contralateral condyle to ensure a fixed size of ROI in the planar image (Fig. 2), mean radiotracer count ratio between the condyles was then calculated. All the image interpretations and ROI analyses were performed on a Xeleris 3 workstation (GE Healthcare)

### 2.5 Image analysis

Nuclear medicine physician with at least 5 years’ experience interpreted the image at the time of imaging as per routine imaging review protocol in the hospital. The scan reports were retrospectively read and categorized by a nuclear medicine physician. The trend of condylar growth was classified as progressive, and relatively stable, regressive categories. Considering the variation of inter–interpreters, we arbitrarily defined ratio value change between scan and subsequent scan of 5% as classification criteria, which means that progressive patients with the positive ratio variation beyond plus 5% during the follow-up scan, while regressive patients with the negative ratio change beyond negative 5%, and the rest were the relatively stable patients.

Finally, to characterize the regressive growth trend toward inactive, we identified the patients with active condylar growth who finally reach to normal range at follow-up scan.

### 2.6 Statistics analysis

Chi-square tests were used in the gender difference analysis. Descriptive feature of variables presents using mean± standard deviation. Statistical significance was set at a 2 tailed *P* value of less than .05 for unknown trend analysis, or a 1 tailed *P* value of less than .05 for known trend analysis. All statistical analyses were performed with SPSS statistical software22.0 (IBM Corp, Armonk, NY).

## 3 Results

### 3.1 Patient characteristics

**Figure 1:**
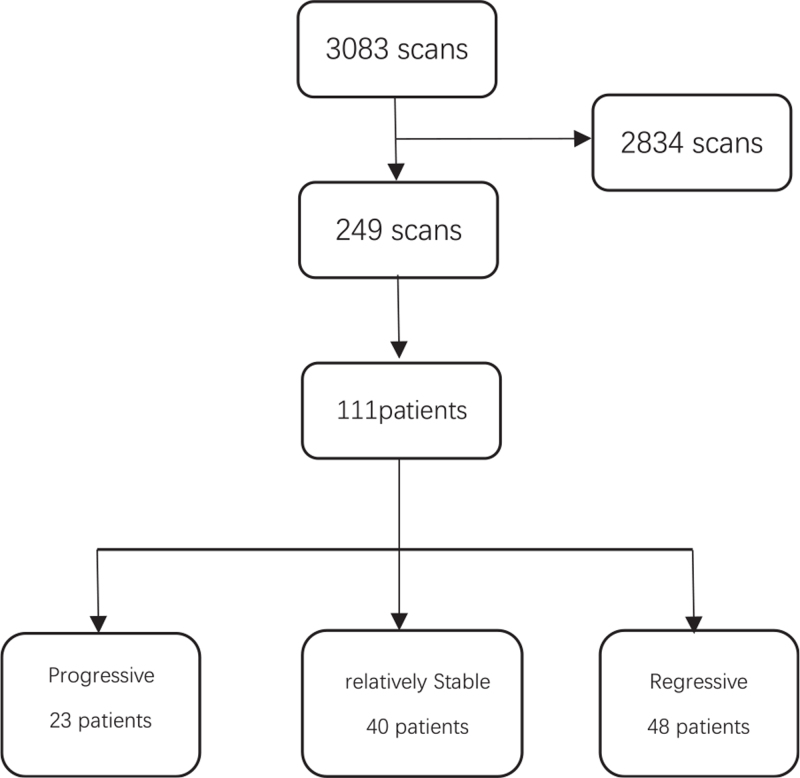
Flow chart of patients selected.

**Figure 2:**
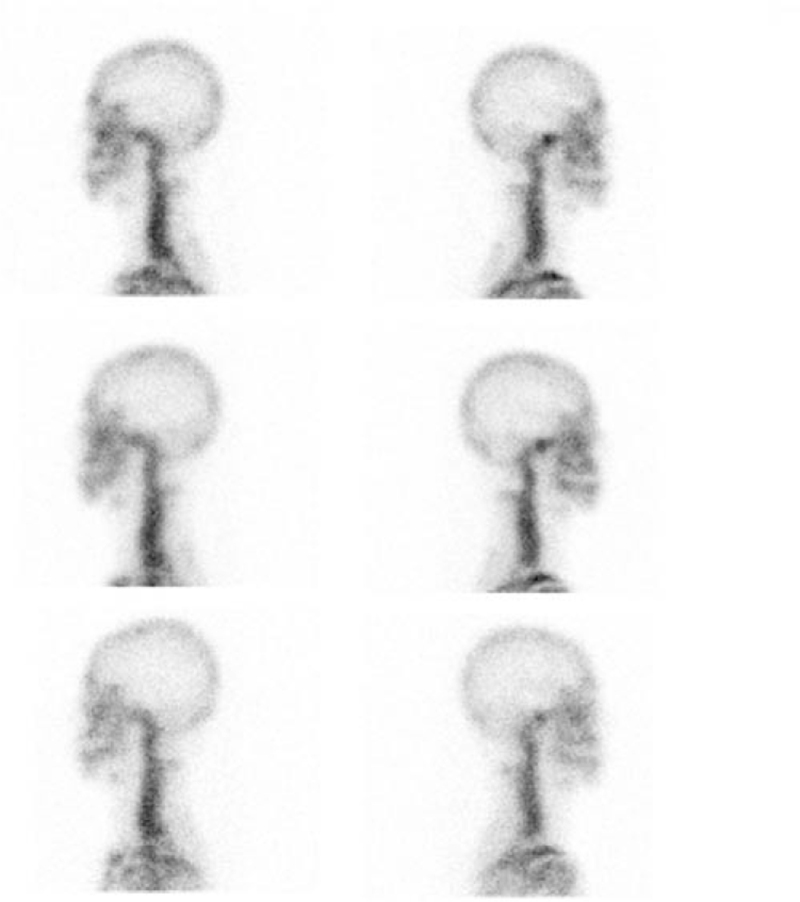
Clinical example 1. Lateral planar imaging of a 19-yr-old male showed regressive trend (from top row down: scans at presentation [right to left ratio 1.26], 15 mo [ratio 1.21], 39 mo [ratio 1.05], respectively).

**Figure 3:**
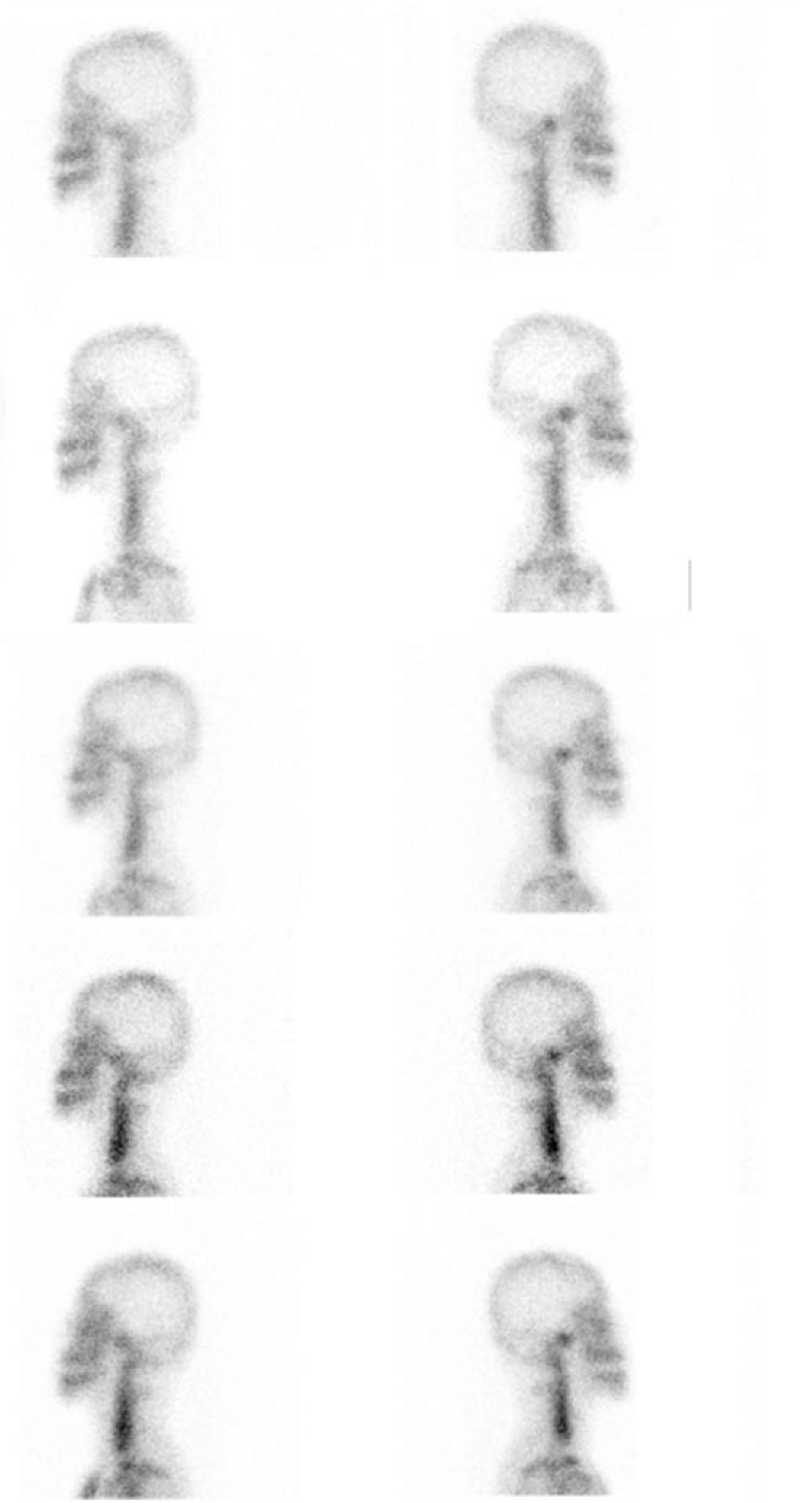
Clinical example 2. Lateral planar imaging of a 19-yr-old female showed relatively stable ratio (from top row down: scans at presentation [ratio 1.21], 12 mo [ratio 1.32], 34 mo [ratio 1.33], 45 mo [ratio 1.29], 55 mo [ratio 1.25], respectively).

A total of 3083 scans of patients suspicious of active UCH who had undergone 99m-Tc-MDP planar bone scintigraphy were included. As showed in Figure 1, of these scans, 249 scans of 111 patients with active UCH who had at least 1 or more follow-up scans were identified. The number of scans per patient ranged 2 to 5 scans. Eighty-seven (78.4%) patients had 1 follow-up scans, 22 (19.8%) patients had 2 follow-up scans (1 example showed in Fig. 2), and 1 patient (0.9%) had 3 follow-up scans and 1 patient (0.9%) had 4 follow-up scans (Fig. 3). The patient demographics have been summarized in Table 1, the age of the patients when they initially underwent 99m-Tc-MDP planar bone scintigraphy was 19.42 ± 2.96 (range 15–36), female patients accounted for 50 (45%), and male 61 (55%), and the age of female patients was (19.42 ± 2.96) and that of male patients was (19.42 ± 2.96). Follow-up periods between first scan and last scan from 1 to 60 months, (16.10 ± 9.09) median is 13 months.

**Table 1:** Patient demographic characteristics.

			Age (yr)	
	Number (%)	Sex ratio (m/f)	mean ± SD	Follow up period (mo)
Group 1	23 (21)	11/12	19.52 ± 2.57	16.91 ± 10.11
Group 2	40 (36)	22/18	19.50 ± 3.65	20.82 ± 12.41
Group 3	48 (43)	28/20	19.58 ± 2.47	16.52 ± 8.14
Total	111	61/50	19.42 ± 2.96	16.10 ± 9.09

Sex ratio difference between groups	
Group1 vs group 2	*P* < .01^∗^
Group 2 vs group 3	*P* = .22
Group1 vs group 3	*P* < .001^∗^
Difference among groups	
Age	*P* = .98
Follow up period	*P* = .36

SD = standard deviation.

∗ Significant difference (*P* < .05)

### 3.2 Categorization of growth trends based on follow-up scans

All patients included in the study were categorized into three subgroups of progressive trend, relatively stable trend, and regressive trend (Figs. 2 and 3).

A total of 23 (21%) patients were classified into the progressive group because of worsened ratio in the follow-up scans. As shown in Table 1, the mean age at the initial scan was 19.52 ± 2.57 years (range: 15–26 years), and the mean follow-up period was 16.91 ± 10.11 months (median: 12 months, range: 2–36 months). There were 12 female patients (52.2%) and 11 male patients (47.8%) included in this group.

Forty (36%) patients were classified as the relatively stable trend for the condyle growth remained active and the ratio variation of follow-up scans fell into the limit, the mean age of patients was 19.50 ± 3.65 years (range: 16.00–36.00 years), the mean follow-up was 20.82 ± 12.41 months (median: 17 months, range: 4.00–60.00 months); this group consisted 18 females (45%) and 22 males (55%).

There were 48 (43%) patients who showed the regressive growth trend with the mean age of 19.58 ± 2.47 years (ranged: 16.00–29.00 years), and the mean follow-up period of 16.52 ± 8.14 years (median: 22 months, range: 2.00–68.00 months); 20 females (42%) and 28 males (58%) included in this group. Of the regressive group, 39 (35%) patients (19 males and 20 females) demonstrated as inactive in the follow-up scan.

### 3.3 Comparison of sex ratio, age, follow-up period among 3 groups

The progressive group showed a higher female ratio compared to both the stable group and the regressive group (*P* < .01, *P* < .001, respectively), which means more female patients in the progressive trend group than those in the stable group or the regressive group, while the stable group and the regressive group have similar sex ratio with more male patients (*P* = .22) (Table 1).

Either the ages or follow-ups of 3 groups analysis was demonstrated no significant difference among the 3 groups (*P* = .98, *P* = .36; respectively).

### 3.4 Correlation between age and ratio difference

Age and values of ratio difference relation analysis showed no strong correlation among 3 subgroups with *r* = −0.102 (*P* = .64) in Group 1, *r* = −0.110 (*P* = .50) in Group 2, *r* = −0.240, (*P* = .10) in Group 3. If combined all subgroups, the overall correlation was not significant either with *r* = −0.107 (*P* = .19) (Table 2).

**Table 2:** Correlation between age and ratio difference.

Group	*r*	*P* value
1	−0.102	.64
2	−0.110	.50
3	−0.240	.10
Total	−0.107	.19

### 3.5 Correlation between age and follow-up period

No strong correlation between age and follow-up period was found subgroups or overall patients with *r* = 0.162 (*P* = .46), *r* = −0.173 (*P* = .28), *r* = −0.016 (*P* = .91), *r* = −0.062 (*P* = .51) in Group 1, Group 2, Group 3, all patients included, respectively (Table 3).

**Table 3:** Correlation between age and follow-up period.

Group	*r*	*P* value
1	0.162	.46
2	−0.173	.28
3	−0.016	.91
Total	−0.062	.51

### 3.6 Correlation between period of follow-up and ratio difference

Period of follow-up and values of ratio difference relation analysis showed Pearson correlation coefficient was 0.549 (*P* < .01) in Group 1, 0.072 (*P* = .66) in Group 2, −0.125, (*P* = .39) in Group 3. And the overall correlation coefficient was −0.075 (*P* = .36) when all groups combined (Table 4).

**Table 4:** Correlation between period of follow-up and ratio difference.

Group	*r*	*P* value
1	0.549	<.01
2	0.072	.66
3	−0.125	.39
Total	−0.075	.36

## 4 Discussion

Our retrospective study showed three growth patterns of a relatively stable, progressive, or regressive trend with bone scintigraph follow-up of UCH of patients with active hyperplasia of condyles.

The etiology of UCH is not clear, but factors such as hormonal disturbance, infection, heredity, metabolic hyperactivity, trauma, intrauterine factors, and hypervascularity may contribute to the UCH development,^[2]^ but somatic mutation of a gene controlling cell growth may not likely be a cause.^[11]^ However, the occurrence of the disease may be at any age, in our study, the mean age of the patients 19.42 ± 2.96 with a range from 15 to 36 years, these data demonstrated that UCH could occur across a large span of age. In our study, the female patients accounted for 50 (45%), and male 61 (55%), which seemly contradicted previous studies that CH, predominantly affect women. A meta-analysis revealed that women develop CH, with 64% (95% confidence interval, 58%–70%; n = 275) of the patients.^[12]^ The discrepancy may be due to patients included in our study only represents a biased sample of patients with active hyperplasia of condyles and with follow-up scans, which may also suggest that male patients more likely require follow-up scans.

Analysis of follow up scans of patients in our study showed mixed growth trends, and we arbitrarily classified them into three groups: the progressive group, the relatively stable group, and the regressive group. Our results showed that only 21% of the patients showed the progressive growth pattern and the remainders (79%) showed stable or regressive growth patterns. Most importantly, just 35% (39 patients) of the total patients reached normal ratio in following scans. A previous study^[13]^ using fluorine-18 fluoride positron emission tomography (PET)-CT scan to invest the change in mandibular condylar hyperactivity over a period a minimum of 1 year showed that similar mean standard uptake value (SUVmax) of the affected condyle (SUVmax T0: 9.18 ± 4.07, SUVmax T1: 9.18 ± 3.88), the authors also noticed the relative isotope uptake in 8 (50%) patients, while the remaining patients had a decrease in relative isotope uptake. However, the author did not further divide the patients into subgroups and analyze due to the smaller sample size (16 patients). To our knowledge, our classification due to the relatively larger patient population is first reported.

Because the status of condylar growth affects the treatment strategy. A corrective osteotomy is performed after cessation of condylar growth, while the high condylectomy may be proper in cases of persistent growth of condyles. Therefore, condylar growth assessment is crucial for the timing of surgery.

More females consisted in the progressive group compared to the other 2 groups (*P* < .01), while the relatively stable or regressive group showed a similar sex ratio with more male patients, the result may reflect that the increased estrogen receptor numbers in the temporomandibular joint of female patients,^[14,15]^ which may be a factor affecting condyle continuate growth. The result was consistent with the previous report that more females affected than males in the active group.^[16]^

However, no significant differences were found in age and follow-up period among those 3 groups. There was no strong correlation between age and ratio difference, and these results may also be explained that the random of occurrence of the growth of condyles among patients, otherwise, it also indicates that it is difficult to predict the growth status of condyles with routinely observed factors such as age or gender, etc.

Using clinical modalities to determine the cessation of mandibular growth is time-consuming and needs at least two measurements 6 to 12 months apart, which may cause unnecessary delay, particularly in children in whom sub-total condylectomy is indicated.^[6,17]^ The finding from the comparison only reveals the status of growth of the past period, it cannot determine the potential of growth in the future.^[17]^ However, there is a report by Chan et al^[16]^ disputed the usage of bone scintigraphy in the determination of the status of condylar growth in UCH, which claimed the sensitivity and specificity of SPECT ranged only between 32.4% and 67.6%, and 36.1% and 78.3%, respectively. It is much less than previously reported sensitivity and specificity with SPECT scans with sensitivity values of between 78% and 98%, and specificity values of between 60% and 95%. Because the authors compared the serial radiographs and clinical photographs at the time of SPECT and that of 1 year later, from present results the growth status determined at the time of the scintigraph could not predict the growth trend of condyles in the following time. with only 21% patients showed the progressive trend, which may correspond to 17% patients showed active growth documented with serial radiographs in the following year of the report. And 35% patients reached truly inactive growth of SPECT in the follow-up scans. While majority of the stably active patient did not show a measurable difference in serial radiographs. The author obviously overestimated the false positive rate of SPECT compared 1 year period of true growth status of condyles.

There are some limitations inherited to the planar bone scintigraphy: first, the methods used in these patients used mean pixel counts method not currently widely accepted maximum pixel counts method,^[17,18]^ maximum pixel counts method may have less variation between observers than mean pixel counts method for that maximum pixel value does not critically depend on the size and exact placement of ROI if maximum value pixel resides within the ROI. However, studies have demonstrated that mean and maximum activities are highly correlated.^[17,19]^ Secondly, planar scintigraphy produces a two-dimensional image, as opposed to SPECT (SPECT/CT) and PET (PET/CT), which produce 3-dimensional images. Since the superimposition of activity from the opposite condyle and adjacent structure will affect the condyle of interest, SPECT had a significantly higher sensitivity than planar scintigraphy in detecting UCH, but the specificity is similar.^[20,21]^ Saridin et al reported that the sensitivity and specificity of planar scans was 67% and 85% compared to 93% and 96% of SPECT,^[18]^ and a recent study reported identical specificity (96%) and slightly more sensitivity of SPECT/CT than planar bone scintigraphy (91% vs 78%) in the evaluation of active UCH.^[21]^ Further studies with SPECT or SPECT/CT were warranted to confirm the results of the current study.

We acknowledge there were a few other limitations to our study. The study was a retrospective study with its biases. The cut-off value was arbitrarily set to classify the patients and needs further studies to confirm its values.

## 5 Conclusions

In conclusion, our retrospective follow-up study showed that three growth patterns of progressive, relative stable, and regressive trend in patients with UCH, about one-fifth of patients showed the progressive trend and less than half patients showed the regressive trend in the follow-up scans, however, there was no significant difference in terms of age and follow-up periods among 3 growth trend groups of the patients.

## Author contributions

**Conceptualization:** Pingan Liu.

**Data curation:** Pingan Liu, Jun Shi.

**Formal analysis:** Pingan Liu.

**Investigation:** Pingan Liu.

**Methodology:** Pingan Liu, Jun Shi.

**Validation:** Jun Shi.

**Writing – original draft:** Pingan Liu, Jun Shi.

**Writing – review & editing:** Pingan Liu.
